# Associations between education and brain structure at age 73 years, adjusted for age 11 IQ

**DOI:** 10.1212/WNL.0000000000003247

**Published:** 2016-10-25

**Authors:** Simon R. Cox, David Alexander Dickie, Stuart J. Ritchie, Sherif Karama, Alison Pattie, Natalie A. Royle, Janie Corley, Benjamin S. Aribisala, Maria Valdés Hernández, Susana Muñoz Maniega, John M. Starr, Mark E. Bastin, Alan C. Evans, Joanna M. Wardlaw, Ian J. Deary

**Affiliations:** From the Centre for Cognitive Ageing and Cognitive Epidemiology (S.R.C., S.J.R., A.P., N.A.R., B.S.A., M.V.H., S.M.M., J.M.S., M.E.B., J.M.W., I.J.D.), Department of Psychology (S.R.C., S.J.R., A.P., J.C., I.J.D.), Brain Research Imaging Centre (D.A.D.,N.A.R., B.S.A., M.V.H., S.M.M., M.E.B., J.M.W.), Neuroimaging Sciences, Centre for Clinical Brain Sciences, and Alzheimer Scotland Dementia Research Centre (J.M.S.), University of Edinburgh; Scottish Imaging Network (S.R.C., D.A.D., N.A.R., B.S.A., M.V.H., S.M.M., M.E.B., J.M.W.), a Platform for Scientific Excellence (SINAPSE) Collaboration, Edinburgh, UK; Department of Neurology and Neurosurgery (S.K., A.C.E.), McConnell Brain Imaging Center, Montreal Neurological Institute, McGill University, Montreal; Department of Psychiatry (S.K.), Douglas Mental Health University Institute, McGill University, Verdun, Quebec, Canada; and Department of Computer Science (B.S.A.), Lagos State University, Lagos, Nigeria.

## Abstract

**Objective:**

To investigate how associations between education and brain structure in older age were affected by adjusting for IQ measured at age 11.

**Methods:**

We analyzed years of full-time education and measures from an MRI brain scan at age 73 in 617 community-dwelling adults born in 1936. In addition to average and vertex-wise cortical thickness, we measured total brain atrophy and white matter tract fractional anisotropy. Associations between brain structure and education were tested, covarying for sex and vascular health; a second model also covaried for age 11 IQ.

**Results:**

The significant relationship between education and average cortical thickness (β = 0.124, *p* = 0.004) was reduced by 23% when age 11 IQ was included (β = 0.096, *p* = 0.041). Initial associations between longer education and greater vertex-wise cortical thickness were significant in bilateral temporal, medial-frontal, parietal, sensory, and motor cortices. Accounting for childhood intelligence reduced the number of significant vertices by >90%; only bilateral anterior temporal associations remained. Neither education nor age 11 IQ was significantly associated with total brain atrophy or tract-averaged fractional anisotropy.

**Conclusions:**

The association between years of education and brain structure ≈60 years later was restricted to cortical thickness in this sample; however, the previously reported associations between longer education and a thicker cortex are likely to be overestimates in terms of both magnitude and distribution. This finding has implications for understanding, and possibly ameliorating, life-course brain health.

Longer education duration is associated with reduced risk of dementia^1^ and cognitive decline^2^ and with several brain MRI measures in older age (such as greater cerebral^3^ or gray matter volume^4^ or ostensibly more favorable white matter macrostructure and microstructure^5,6^). However, the current understanding of which brain measures characterize the putative benefits of education is hampered by analyses of isolated MRI biomarkers, sometimes small sample sizes, and the absence of an important potential confounder that we address here: preexisting cognitive ability.

Most recently, longer education was reportedly associated with a thicker cortex across several loci,^7^ which was interpreted as evidence that education may increase brain or cognitive reserve: “Our findings suggest the protective effect of education on cortical thinning in…older individuals.”^7(p806)^ Such causal statements are often seen,^1–6^ but are they warranted? Specifically, the contribution of education level to brain measures in older age, beyond preexisting cognitive differences that are present from earlier development, is unknown. Intelligence at age 11 accounts for some variation in cortical thickness^8^ and white matter macrostructure^9,10^ and microstructure^11^ in older adulthood. Educational and cognitive ability are substantially phenotypically^12^ and genetically^13,14^ correlated. Thus, early-life intelligence may partly confound education-brain associations; more intelligent children tend to stay in education longer^15^ and have brains that ultimately age well. Alternatively, these 2 predictors might provide incremental information about older-age brain structure. In the current study, we adopt an analytic approach similar to that used by Kim et al.^7^ to examine associations between education, cortical thickness, and other MRI measures among older adults, conducting analyses before and after accounting for age 11 intelligence.

## METHODS

### Participants.

In June 1947, almost all Scottish school children born in 1936 took a validated intelligence test (Moray House Test No.12 [MHT]) at ≈11 years of age. Schoolchildren at the time entered formal education at 5 years of age. They then spent 7 years in primary school, where they were taught mostly English and arithmetic without subject specialization. The transition to secondary education took place at 11 or 12 years of age. Children were often streamed by ability, received different curricula, and stayed at school thereafter for a different number of years. Therefore, IQ at age 11 represents a measure of cognitive ability before the time at which variation in education duration and topics took effect. Indeed, the MHT was deliberately administered at age 11 to capture cognitive data before the transition from primary to secondary school (the earliest school-leaver in the current sample was 14 years old and thus experienced ≈50% more education between the MHT and leaving school) to avoid, as far as possible, the effect of educational differences on cognitive ability test scores.

Between 2004 and 2007, 1,091 (543 female) community-dwelling older adults, most of whom took the MHT at age 11, were recruited into the Lothian Birth Cohort 1936 study.^16^ Approximately 3 years later (at ≈73 years of age), 866 (79%) returned for a second wave of follow-up testing, including an optional MRI brain scan that was undertaken by 728, yielding 681 participants with usable MRI data.^17^ Main reasons for nonattendance at this second wave were death or self-assessment of inability to participate. Early termination of scan and movement artifacts were the main reasons for the loss of MRI data. Of these, 617 also had age 11 MHT scores, self-reported years of full-time education, and contemporaneous information on health during the medical interview: self-reported history of hypertension, hypercholesterolemia, diabetes mellitus, and body mass index, which we refer to collectively as vascular risk factors.^18^ All 617 participants had a Mini-Mental State Examination^19^ score of >24 and reported no diagnosis of dementia.

### Standard protocol approvals, registrations, and patient consents.

The Multi-Centre Research Ethics Committee for Scotland (MREC/01/0/56) and Lothian Research Ethics Committee (LREC/2003/2/29) approved the use of the human participants in this study. All participants provided written informed consent, and these forms have been kept on file.

### Brain MRI acquisition.

Full details of the whole-brain MRI structural and diffusion protocol are available open access.^17^ Briefly, T2-, T2*-, and fluid-attenuated inversion recovery–weighted axial volumes, a high-resolution coronal T1-weighted volume (1 × 1 × 1.3 mm), and diffusion imaging were acquired on the same 1.5T GE Signa Horizon HDx clinical scanner (General Electric, Milwaukee, WI). The diffusion MRI protocol consisted of 7 T2-weighted and a set of diffusion-weighted (b = 1000 s/mm^2^) axial single-shot spin-echo echo-planar volumes acquired with diffusion gradients applied in 64 noncollinear directions (dimensions 2 × 2 × 2 mm).

### Cortical thickness measurement.

We measured cortical thickness using the CIVET image processing pipeline developed at the Montreal Neurological Institute.^20,21^ CIVET performs the following steps^22^: (1) registration of T1-weighted volumes to an age-specific template; (2) bias field correction; (3) brain extraction; (4) segmentation of gray and white matter and CSF; (5) definition of cortical thickness at 81,924 vertices (the perpendicular distance between gray and white matter surfaces) across the cortex via the t-link metric; (6) inverse of registration at step 1 for cortical thickness measurements in the native space of each participant; and (7) smoothing with a 20-mm kernel.

We visually inspected the output blinded to all participant characteristics. Approximately 10% of participants failed CIVET processing because of poor scan quality/motion artifact, and their cortical thickness maps were excluded from the analysis. The final number of 548 participants included in the present analysis includes only those who passed visual inspection.

### Brain volumetry.

Intracranial volume (ICV) and total brain volume were measured with a validated semiautomated multispectral fusion method using T1-, T2-, T2*, and fluid-attenuated inversion recovery–weighted sequences.^23^ ICV included all structures and CSF inside the dura, where the lower limit was the axial slice immediately inferior to the limit of the cerebellar tonsils on or above the superior tip of the odontoid process. Brain atrophy was computed as total brain volume as a proportion of ICV. All segmented images were visually examined by researchers blinded to participant characteristics for accuracy on anonymized scans to correct errors.^24^

### Tractography.

After preprocessing of the diffusion data (brain extraction, removal of bulk patient motion and eddy current–induced artifacts), parametric maps of fractional anisotropy (FA) were generated for every participant with freely available tools in FSL (FMRIB, Oxford, UK: http://www.fmrib.ox.ac.uk). Tract-averaged FA values were determined for 12 tracts of interest (genu and splenium of corpus callosum; bilateral anterior thalamic radiations, cingulum bundles, arcuate, uncinated, and inferior longitudinal fasciculi) using probabilistic neighborhood tractography with the BedpostX/ProbTrackX algorithm,^25^ which offers reproducible segmentation of major white matter pathways^26,27^ (http://www.tractor-mri.org.uk). Visual inspection of the tract masks was conducted to ensure that they were anatomically plausible. Segmentations that exhibited aberrant or truncated pathways were excluded. To reflect the relatively high degree of shared variance in diffusion characteristics across white matter tracts,^28^ we derived a measure of general FA (gFA) from the first unrotated principal component of FA across all pathways, explaining 40% of the variance. A total of 333 participants had list-wise complete data; the number of specific tracts available is shown in table e-1 at Neurology.org.

### Statistical analysis.

All statistical analyses were performed in R version 3.03 (https://www.r-project.org) except for cortical thickness analyses, which were performed with the SurfStat MATLAB toolbox (http://www.math.mcgill.ca/keith/surfstat) for Matrix Laboratory R2014a (The MathWorks, Inc, Natick, MA). We assessed associations of years of education and age 11 IQ with brain MRI measures in older age using 2 models. In model 1, only sex, years of education, and vascular risk factors were independent variables. In model 2, age 11 IQ was an additional independent variable. Age in days at MRI scanning was also entered into both models to account for any residual effect of age on brain structure. Initially, average cortical thickness atrophy and gFA were analyzed. Adding an interaction term between education and age 11 IQ addressed whether both might be important for later-life brain status beyond their individual main effects. Next, we conducted region-specific analysis of cortical thickness. Models 1 and 2 were tested for each vertex across the cortical mantle both with and without correcting for ICV. Finally, we ran models 1 and 2 for tract-averaged FA in each of the 12 white matter fasciculi. We set α at 0.05, and the false discovery rate (FDR) was used to correct for multiple comparisons (Q values are reported in figures for vertex-wise regressions^29^). Models were examined for multicollinearity with the variance inflation factor (VIF in the HH package for R). In a supplementary analysis, we matched participants with less (9 or 10 years) education with those who had more education (≥11 years) on the basis of propensity scores for age 11 IQ, age, and sex using the nonrandom package for R (matches were made within 0.05 SD of the logit of the propensity score). If longer education conferred a cerebral advantage beyond preexisting intelligence differences, this would be indicated by a significant difference between groups that were well matched on age 11 IQ (rather than using a statistical control, as in the main analysis).

## RESULTS

Descriptive statistics of the study participants are given in table 1. Bivariate (uncorrected) correlations among study variables are reported for illustrative purposes in table e-2. Years of education ranged from 9 to 14 years. A higher age 11 IQ and more years of education were significantly correlated (*r* = 0.416, *p* < 0.001).

**Table 1:**
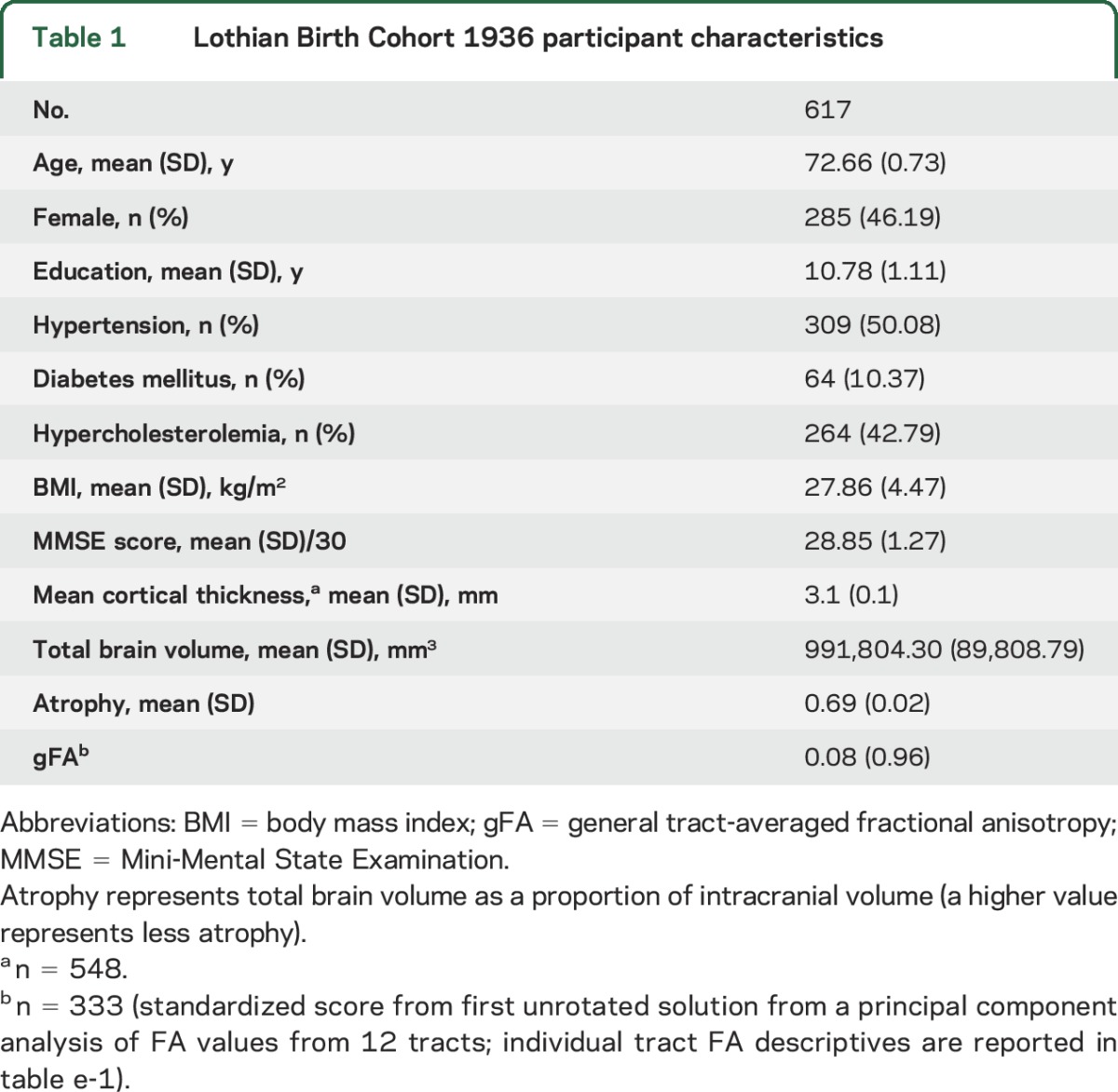
Lothian Birth Cohort 1936 participant characteristics

### Associations of education and age 11 IQ with cortical thickness.

The associations between education and age 11 IQ and average thickness of the entire cortical mantle based on regression analyses are shown in table 2 (standardized β values reported throughout). Considering education alone (model 1), more years were associated with a generally thicker cortex. The relationship between education and average cortical thickness was attenuated by ≈23% when age 11 IQ was included (model 2), although age 11 IQ was not significant in the model. We found no significant interactions between education and age 11 IQ for cortical thickness (β = −0.002, *p* = 0.940), atrophy (β = 0.007, *p* = 0.752), or either general (β = −0.015, *p* = 0.665) or tract-specific (β_absolute_ < 0.002, *p* > 0.117) FA.

**Table 2:**
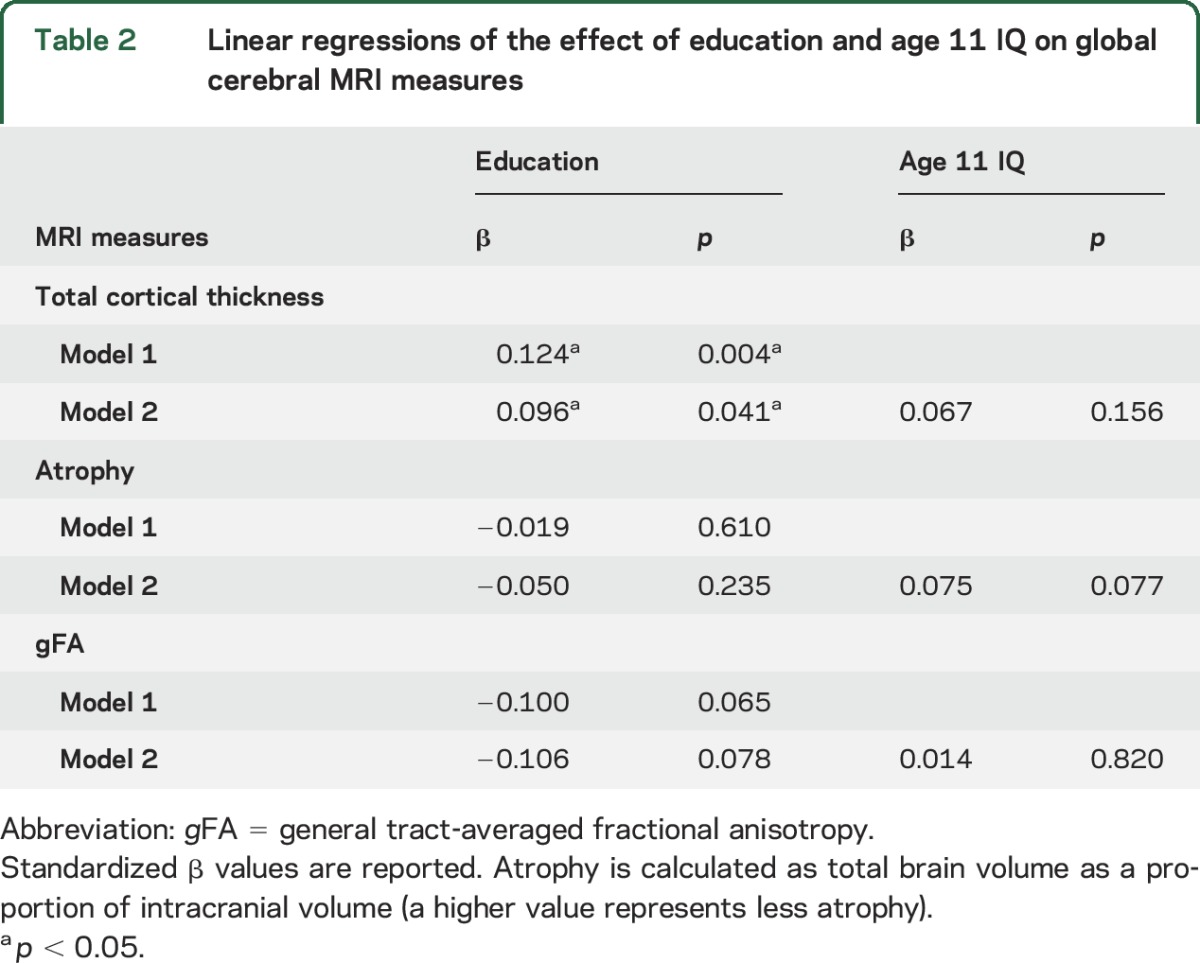
Linear regressions of the effect of education and age 11 IQ on global cerebral MRI measures

**Figure 1:**
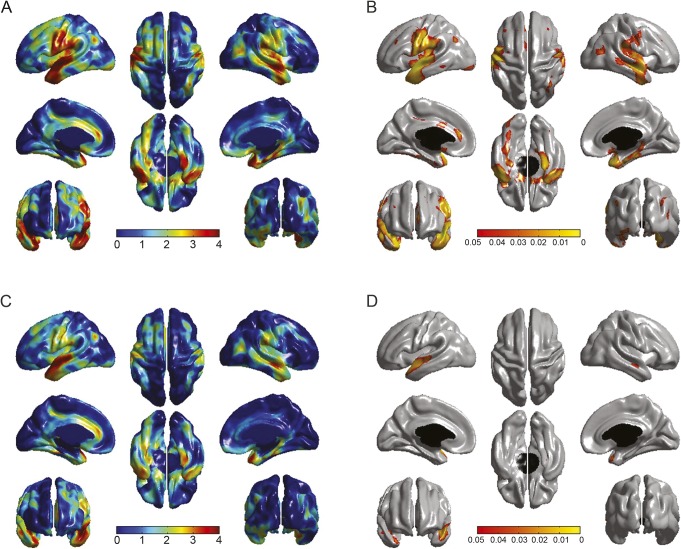
Associations between years of education (EDU) and cortical thickness, before and after age 11 IQ correction (EDU,adj 11yrIQ) Uncorrected (*t* maps, A) and false discovery rate–corrected (*q* maps, B) associations between cortical thickness and education (top) and cortical thickness and education with adjustment for age 11 IQ (C and D). The extent of false discovery rate–corrected significant positive associations between cortical thickness and education is reduced by >90% with adjustment for age 11 IQ. Both models are controlled for vascular risk factors.

**Figure 2:**
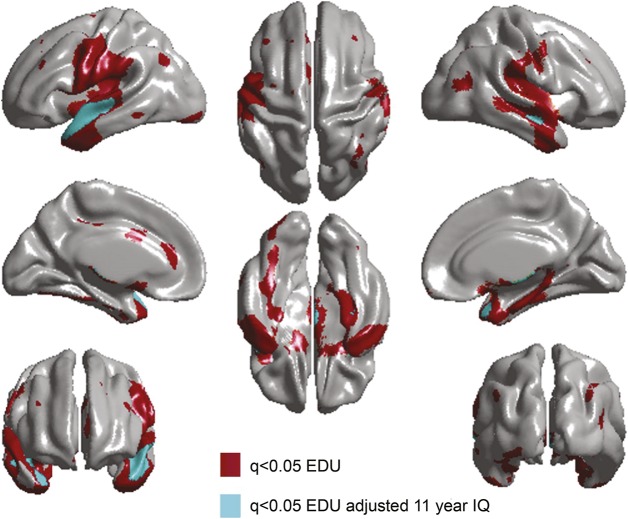
Overlap and mismatch of cortical thickness–education (EDU) associations, before and after age 11 IQ correction (EDUadj 11yrIQ) False discovery rate–corrected significant (q value >0.05) positive associations between cortical thickness and education and between cortical thickness and education with adjustment for age 11 IQ.

Longer education was associated with a thicker cortex in bilateral temporal, medial frontal, parietal, somatosensory, and motor cortices (figure 1, top). Entering age 11 IQ into the analysis (model 2; figure 1, bottom) reduced the extent of the FDR-corrected significant associations by 90.6% (from 12,820 to 1,209 significant vertices). Unique effects of longer years of education on a thicker cortex remained in only the superior temporal regions, bilaterally. The spatial extent of this confounding is illustrated in figure 2. Associations between age 11 IQ and vertex-wise cortical thickness in the Lothian Birth Cohort 1936 (in the absence of education and health covariates) were reported previously.^8^ We also ran the same analysis including ICV as a covariate, but its inclusion had no impact on our findings (figure e-1).

### Contributions of education and age 11 IQ to other cerebral measures in older age.

Neither education nor age 11 IQ was significantly associated with global atrophy or with gFA at age 73 (table 2). Associations between years of education and tract-averaged FA in model 1 were consistently negative but largely null. Only 1 of 12 tests was significant, specifically for genu of the corpus callosum, and it remained significant when age 11 IQ (model 2) was included and survived FDR correction (table e-3). Throughout the analysis, all models were found to exhibit acceptably low multicollinearity (variance inflation factor < 2.262 across all covariates tested in each model^30^).

The results above are corroborated by our supplementary propensity score–matched analyses, reported in table e-4 and figure e-2.

## DISCUSSION

The patterns of frontal, temporal, and parietal areas where greater cortical thickness is associated with longer education are similar to those found previously,^7^ ostensibly corroborating those authors’ interpretation that longer education confers greater thickness across distributed cortical sites. They further interpreted their findings as evidence for education increasing resistance to brain structural loss from aging, as opposed to a fixed cerebral advantage in these regions. However, when we included prior childhood cognitive ability in our model, estimates of the size and cortical distribution of this effect were markedly reduced. Remaining significant effects were confined to bilateral anterior portions of the superior temporal cortex. This is a region with strong links to acquiring and recall of semantic knowledge,^31,32^ and it is posited as an amodal hub that integrates modality-specific information from a more distributed semantic network.^33^ Alternatively, diminished accuracy of anterior temporal cortical thickness estimates due to partial volume effects could explain these residual associations, such that the remaining associations may be artifactual and spurious. Our supplementary analysis using propensity matching showed no significant differences in cortical thickness at any point on the mantle, although this could be attributable to the diminished power to detect this small effect (visible in the anterior temporal lobe on the uncorrected t maps). Our own interpretation of these data is that any effect of years of education on cortical thickness is likely to be an overestimate when not considered along with preexisting differences in cognitive ability. The finding that more education, regardless of preexisting differences in age 11 IQ, is limited to greater cortical thickness in a region that allows flexible integration of semantic information affords a plausible interpretation but requires replication and exploration with additional study designs.

How the reported associations pertain to different theories about cognitive and brain reserve in aging is not possible to assess within the current cross-sectional framework. A previous study^7^ analyzed education and cortical thickness across a wide age range, a feature that the current cohort lacks. That study found that the strength of the association between cortical thickness and education was modestly (but significantly) larger with greater age, which those authors interpreted as support for increased cognitive reserve or plasticity on age-related cortical thinning. In the current data, we are unable to comment on when (or how) individual differences in cortical thickness manifest or alter as a function of education and the rate at which any change in cortical thickness might be taking effect. Future longitudinal data are required to examine how the association of education with the cortex (perhaps with a focus on anterior temporal regions) changes during development and throughout the life course to more reliably parse apart the cause and effect of these relationships and their putative benefit for brain and cognitive aging.

In addition to cortical thickness, we analyzed associations of age 11 IQ and education with other MRI indexes. Our findings were essentially null for associations between education or age 11 IQ with global atrophy and gFA. Although this might indicate that any relationship between education and brain structure in older age is restricted to cortical thickness, we found a specific effect of longer education on lower FA in genu of the corpus callosum that survived FDR correction. This finding was unexpected, given that FA shows cross-sectional associations and longitudinal declines with age (which are thought to partly reflect older-age–related degradation in axonal myelin and decreased information transfer efficiency^34,35^) and that, in this sample, declines in FA and cognitive ability are coupled.^36^ However, diffusion characteristics are also influenced by several other microstructural properties of white matter, and alterations to these are highly dynamic into early adulthood. Development and refinement of functional circuits (including axonal pruning and increases in axon diameter) could plausibly drive negative education-FA associations^37^ and genu of the corpus callosum changes most dramatically during childhood and early development.^38^ Thus the cross-sectional differences in FA reported here might reflect a small but detectable influence of education in earlier life. This might suggest that concretely viewing high FA as optimal would be to ignore the multiple life-course influences on development and learning, as well as degeneration. Such an interpretation is highly speculative, however, and requires further investigation. This also emphasizes the importance of longitudinal designs for maximizing the value of water diffusion measures when studying the determinants and outcomes of white matter changes in older age.

The study has limitations. First, although the lag between childhood IQ, education, and brain is rare and spans >60 years, other factors that might affect brain development and aging across this span were not captured by the current study. Nevertheless, this lag is highly valuable for examining the covariance of education and prior intelligence with brain structure many years later. Second, each of our measures is cross-sectional. Thus, while it may be tempting to suggest that both education and age 11 IQ contribute to brain reserve (the degree of susceptibility to age-related structural change), the current data are insufficient to inform questions on the aging process per se; rather, we can only comment on extant brain differences in older age. This limitation is partly instantiated by associations reported here and previously^7^ between education and cortical thickness (without correction for age 11 IQ) in the motor and sensory strips. The implication of these regions in education and intelligence might be due to shared genetic and life-course factors with frontal and temporal regions rather than the fact that they directly contribute to intelligence or are influenced by education. Third, we measured only number of years of education; educational experience, quality, specific subjects learned, or measures of attainment may have different effects on cerebral tissue types or loci. Education also showed lower variance (SD = 1.11 years) than in previous work,^7^ yet this relatively low variance still explained significant variation in cortical thickness at comparable loci. Furthermore, the cortical thickness pipeline used here allowed close comparison with previous work,^7^ although future studies may make alternative processing decisions such as adjusting the smoothing kernel to attempt finer-grained analyses (but see the work by Lerch and Evans^39^) or selecting alternative processing pipelines.

These findings indicate that only a modest amount of variation in older-age cortical thickness (but not global atrophy or white matter FA) is related to years of education. However, the spatial distribution of the education effect on cortical thickness in older age is likely to be overestimated if prior cognitive ability is not considered. The loci of remaining effects in the anterior temporal cortices are plausible on the basis of prior evidence for the functional role of these regions as an integrative hub for semantic knowledge. To address the value of education and age 11 IQ for brain reserve adequately, longitudinal data are necessary to test the interplay of these early-life predictors on trajectories of cerebral aging.

## Supplementary Material

Data Supplement

## GLOSSARY

FA: fractional anisotropy
FDR: false discovery rate
gFA: general fractional anisotropy
ICV: intracranial volume
MHT: Moray House Test No.12
