# Supplementary material for: Associations between education and brain structure at age 73 years, adjusted for age 11 IQ
Source: Neurology. 2016 Oct 25;87(17):1820–6. doi: 10.1212/WNL.0000000000003247 (PMC5089529; doi:10.1212/WNL.0000000000003247)
Supplement: Data Supplement [file supp_87_17_1820__index.html]

Associations between education and brain structure at age 73 years, adjusted for age 11 IQ — Data Supplement 

# Associations between education and brain structure at age 73 years, adjusted for age 11 IQ

## Data Supplement

**Neurology® data supplements are not copyedited before publication. Published editorials and translations have been copyedited.  
 © 2016 American Academy of Neurology.  
  
 Files in this Data Supplement:**

- Data Supplement - Microsoft Word file
