# Supplementary material for: Associations between education and brain structure at age 73 years, adjusted for age 11 IQ
Source: Neurology. 2016 Oct 25;87(17):1820–6. doi: 10.1212/WNL.0000000000003247 (PMC5089529; doi:10.1212/WNL.0000000000003247)
Supplement: Data Supplement [file supp_WNL.0000000000003247_Supplemental_Data.docx]

**NEUROLOGY/2015/707430**

**Supplemental Material**

Table e-1. Descriptive statistics for tract FA.

| Tract | Mean FA | SD | N |
| --- | --- | --- | --- |
| Genu | 0.41 | 0.05 | 579 |
| Splenium | 0.49 | 0.07 | 594 |
| L Arc | 0.45 | 0.04 | 573 |
| R Arc | 0.43 | 0.04 | 523 |
| L ATR | 0.32 | 0.03 | 497 |
| R ATR | 0.33 | 0.03 | 575 |
| L Cingulum | 0.44 | 0.05 | 575 |
| R Cingulum | 0.39 | 0.05 | 581 |
| L Uncinate | 0.33 | 0.03 | 511 |
| R Uncinate | 0.33 | 0.03 | 562 |
| L ILF | 0.40 | 0.05 | 595 |
| R IFL | 0.38 | 0.05 | 595 |

*Note*. Arc = arcuate fasciulus, ATR = anterior thalamic radiation, ILF = inferior longitudinal fasciculus, FA = fractional anisotropy.

Table e-2. Associations between education, age 11 IQ and global cerebral measures.

|  | Education | Age 11 IQ | Atrophy | CtxThk |
| --- | --- | --- | --- | --- |
| Education | - |  |  |  |
| Age 11 IQ | .416^c^ | - |  |  |
| Atrophy | .014 | .105^b^ | - |  |
| CtxThk | .121^b^ | .111^b^ | .102^a^ | - |
| *g*FA | -.079 | .009 | .258^c^ | .243^c^ |

*Note.*  ^a^ *p* < .05, ^b^ *p* < .01, ^c^ *p* < .001. Atrophy = total brain volume as a proportion of intracranial volume, CtxThk = average cortical thickness, *g*FA = tract-averaged fractional anisotropy. Bivariate associations are provided here for illustrative purposes, but we note that the association between Education and Age 11 IQ has previously been reported in this sample^1,2^ and in^3^ as a path in an SEM analysis. Correlations between intracranial volume, total brain volume (not corrected for ICV) and Age 11 IQ have been reported in^2,4^. The association between *g*FA and Age 11 IQ has previously been reported in^5^ as a path in an SEM analysis.

Table e-3. Linear regressions of the effect of education and age 11 IQ on global cerebral MRI measures.

| **MRI measures** |  | **Education** | | **Age 11 IQ** | |
| --- | --- | --- | --- | --- | --- |
|  |  | ***β*** | ***p*** | ***β*** | ***p*** |
|  |  |  |  |  |  |
| **Genu FA** | **Model 1** | **-.127** | **.001** |  |  |
|  | **Model 2** | **-.136** | **.002** | .019 | .678 |
| **Splenium FA** | **Model 1** | .013 | .475 |  |  |
|  | **Model 2** | .005 | .579 | .008 | .864 |
| **Left Arcuate FA** | **Model 1** | -.062 | .309 |  |  |
|  | **Model 2** | -.078 | .083 | .040 | .379 |
| **Right Arcuate FA** | **Model 1** | -.016 | .351 |  |  |
|  | **Model 2** | -.023 | .257 | -.008 | .859 |
| **Left ATR FA** | **Model 1** | -.050 | .243 |  |  |
|  | **Model 2** | **-.093** | **.029** | .076 | .113 |
| **Right ATR FA** | **Model 1** | .028 | .475 |  |  |
|  | **Model 2** | -.039 | .371 | **.118** | **.007** |
| **Left Cingulum FA** | **Model 1** | .015 | .706 |  |  |
|  | **Model 2** | .001 | .978 | .004 | .925 |
| **Right Cingulum FA** | **Model 1** | -.014 | .727 |  |  |
|  | **Model 2** | -.009 | .830 | .001 | .990 |
| **Left Uncinate FA** | **Model 1** | -.027 | .525 |  |  |
|  | **Model 2** | -.069 | .152 | .089 | .065 |
| **Right Uncinate FA** | **Model 1** | -.017 | .671 |  |  |
|  | **Model 2** | -.055 | .222 | .070 | .123 |
| **Left ILF FA** | **Model 1** | -.058 | .140 |  |  |
|  | **Model 2** | **-.098** | **.025** | .033 | .451 |
| **Right ILF FA** | **Model 1** | -.001 | .979 |  |  |
|  | **Model 2** | -.003 | .946 | .000 | .996 |

*Note.* Standardised betas are reported. Bold type indicates *p* < .05. Only associations between Education and Genu FA survive FDR correction.

Table e-4. Lothian Birth Cohort 1936 participant characteristics – split into two groups of low (<11 years) and high (≥11 years) of education, propensity score-matched on age 11 IQ, sex and age.

|  |  |  | Low | High | difference *p*^†^ |
| --- | --- | --- | --- | --- | --- |
| N |  |  | 197 | 197 |  |
| Age 11 IQ |  |  | 102.83 (11.81) | 104.37 (10.88) | 0.180 |
| Age, M (SD), yrs | M (SD) years |  | 72.58 (0.74) | 72.63 (0.74) | 0.561 |
| Female | n (%) |  | 99 (50.25%) | 98 (49.75%) | 0.920 |
| Education | M (SD) years |  | 9.95 (0.22) | 11.84 (0.81) | <0.001 |
| Hypertension | n (%) |  | 97 (49.24%) | 98 (49.75%) | 0.920 |
| Diabetes | n (%) |  | 26 (13.20%) | 15 (7.61%) | 0.070 |
| Hypercholesterolemia | n (%) |  | 76 (38.58%) | 91 (46.19%) | 0.126 |
| BMI | M (SD) kg/m^2^ |  | 27.94 (4.24) | 27.64 (4.20) | 0.485 |
| MMSE | M (SD) /30 |  | 28.93 (1.22) | 29.05 (1.12) | 0.323 |
| Mean Cortical Thickness | M (SD) mm |  | 3.11 (0.14) ^a^ | 3.13 (0.14)^b^ | 0.124 |
| Atrophy | M (SD) |  | 0.69 (0.02) | 0.69 (0.02) | 0.090 |
| *g*FA |  |  | 0.23 (0.91)^c^ | -0.02 (0.97)^d^ | 0.066 |
| Genu FA |  |  | 0.42 (0.04)^e^ | .40 (0.05)^e^ | 0.005 |

*Note.* BMI = body mass index, MMSE = Mini Mental State Exam, *g*FA = general tract-averaged fractional anisotropy (standardised score from first unrotated solution from a PCA of FA values from 12 tracts). Atrophy represents total brain volume as a proportion of intracranial volume. ^a^ n = 173, ^b^ n = 174, ^c^ n = 105, ^d^ n = 99, ^e^ = 183, ^†^ = t-test, unless binary data, in which case chi squared test was used.Supplemental Figure Captions

Figure e-1

Title: Associations between years of education and cortical thickness, before and after age 11 IQ correction – with additional correction for intracranial volume.


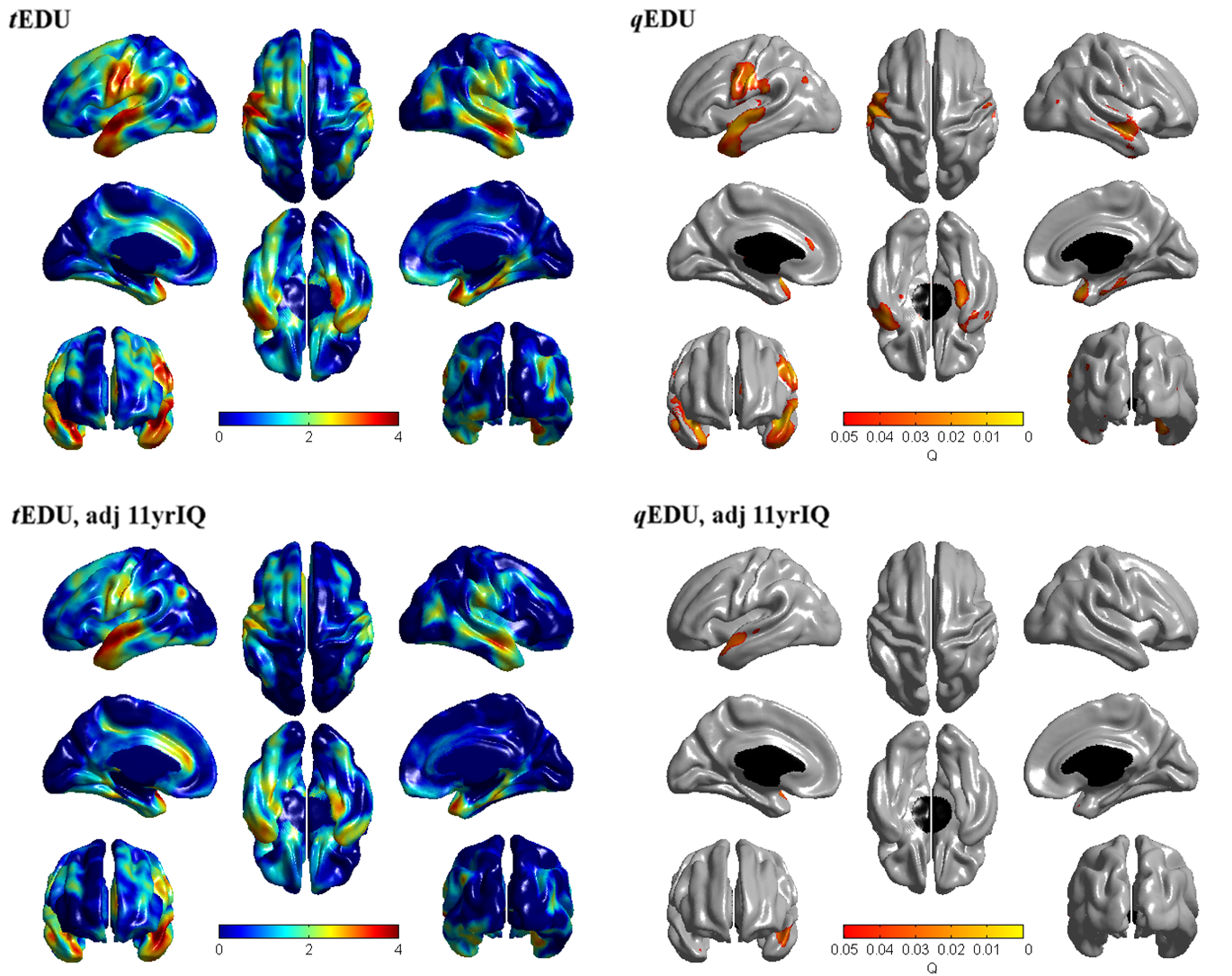


Caption: Uncorrected (*t* maps - left) and False Discovery Rate corrected (*q -* right) associations between cortical thickness and education (top panel) and cortical thickness and education adjusting for age 11 IQ (bottom panel). The extent of FDR corrected significant positive associations between cortical thickness and education is reduced by >90% when adjusting for age 11 IQ. Both models are controlled for vascular risk factors and intracranial volume. Correction for age 11 IQ results in a reduction of 91.29% (4960 to 432) in the number of significant vertices.

Figure e-2

Title: Cortical thickness differences between high and low education groups, propensity score-matched for age 11 IQ, age and sex.


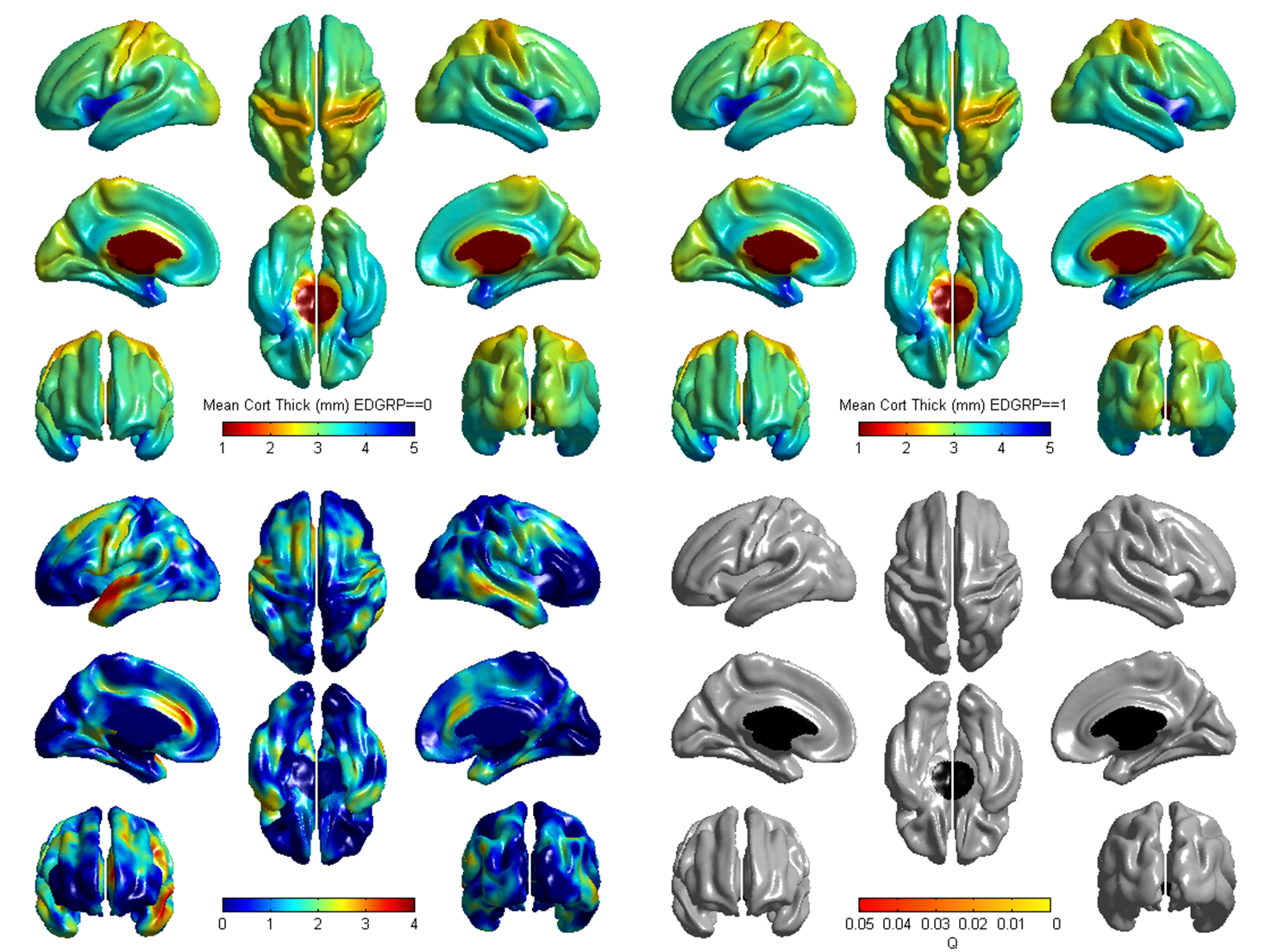


Caption: Mean cortical thickness maps for both groups are shown on the top row (top left = 9-10 years of education, top right = 11+ years of education). Group differences are shown on the bottom row (uncorrected *t* maps = bottom left, False Discovery Rate corrected *q* values = bottom right).

Supplemental References

1. Ritchie SJ, Bates TC, Der G, Starr JM, Deary IJ. Education is associated with higher later life IQ scores, but not with faster cognitive processing speed. Psych Aging 2013; 28:515-521.
2. Booth T, Royle NA, Corley J, Gow AJ, Valdés Hernández MC, Muñoz Maniega S, Ritchie SJ, Bastin ME, Starr JM, Wardlaw JM, Deary IJ. Association of allostatic load with brain structure and cognitive ability in later life. Neurobiol Aging 2015; 36:1390-1399.
3. Ritchie SJ, Bates TC, Deary IJ. Is education associated with improvements in general cognitive ability, or in specific skills? Dev Psychol 2015; 51:573-582.
4. Royle NA, Booth T, Valdés Hernández MC, Penke L, Murray C, Gow AJ, Muñoz Maniega S, Starr JM, Bastin ME, Deary IJ, Wardlaw JM. Estimated maximal and current brain volume predict cognitive ability in old age. Neurobiol Aging 2013; 34:2726-2733.
5. Penke L, Muñoz Maniega S, Bastin ME, Valdés Hernández MC, Murray C, Royle NA, Starr JM, Wardlaw JM, Deary IJ. Brain white matter tract integrity as a neural foundation for general intelligence. Mol Psych 2012; 17:1026-1030.
